# Full-day sleep pattern analysis in common mental disorders: Leveraging highly discrepant recordings from two consumer tracking devices

**DOI:** 10.1371/journal.pone.0346876

**Published:** 2026-04-09

**Authors:** Óscar Jiménez Rama, Antonio Artés, Enrique Baca-García, Jorge López-Castromán

**Affiliations:** 1 Department of Signal Theory and Communications, Carlos III University, Madrid, Spain; 2 Evidence Based Behavior (eB2), Madrid, Spain; 3 Instituto de Investigación Sanitaria Fundación Jiménez Díaz, Madrid, Spain; Universidad Autónoma de Madrid, Madrid, Spain; 4 Departamento de Psiquiatría, Hospital Rey Juan Carlos Móstoles, Madrid, Spain; 5 Departamento de Psiquiatría, Universidad Autónoma de Madrid, Madrid, Spain; 6 Department of Psychiatry, Hospital Universitario Fundación Jiménez Díaz, Madrid, Spain; 7 Universidad Católica del Maule, Talca, Chile; 8 Department of Psychiatry, Hospital Universitario General de Collado Villaba, Madrid, Spain; 9 Departamento de Psiquiatría, Hospital Universitario Infanta Elena Valdemoro, Madrid, Spain; 10 Centro de Investigación Biomédica en Red de Salud Mental, Madrid, Spain; CIBERSAM, Research Group CB/07/09/0025, Madrid, Spain; 11 Department of Psychiatry, Radiology, Public Health, Nursing and Medicine, University of Santiago de Compostela, Santiago, Spain; Helwan University Faculty of Engineering, EGYPT

## Abstract

Sleep and circadian rhythm disruptions are increasingly studied through consumer sleep-tracking devices, both in research and in real-world contexts. These devices offer a unique perspective on mental health, given the strong connection between sleep disturbances and Common Mental Disorders (CMD). In this study, we sought to identify and characterize abnormal sleep behaviors by examining discrepancies between two complementary sleep-tracking devices. Rather than treating inter-device disagreement as measurement noise, we interpreted it as a potential behavioral signal. This approach uncovered six statistically robust outlier patterns in sleep health that were interpretable and clinically relevant. These patterns span a full 24-hour window—including nocturnal, diurnal, and peri-sleep activities—thus providing a holistic view of sleep-related behavior. We analyzed data from 149 patients (72% woman), ranging from 18 to 71 years old, and diagnosed with non-severe CMD over a period of three months. At the end, 4,824 days of sleep recordings were collected from two devices: a less accurate wristband tracker (W) and a more precise sleep-tracking mat (M). Using k-means clustering on high-discrepancy recordings (>5 hours), we identified six robust patterns of full-day sleep behavior that exhibited consistency at the individual user level, suggesting an origin in the patient’s behavior rather than random noise. To further validate these clusters, we integrated additional behavioral metrics in the analysis such as daily step distribution or smartphone usage as indicators of physical or social activity. By leveraging device discrepancies, we revealed several sleep patterns of potential clinical relevance—indicative of oversleeping, unintended sleep onset outside the bed, or atypical sleep-wake cycles. These findings highlight the potential of passive sleep monitoring to support early detection of pathological changes (e.g., depressive episodes) and to inform clinical decisions by identifying behavioral side effects of treatment.

## Introduction

Sleep quality and its patterns have been widely correlated with both mental [1] and physical [2] health in medical research, manifesting short [3] and long term [4] impacts, particularly during adolescent development [5]. For example, sleep disorders are frequently associated with Common Mental Disorders (CMD), which are prevalent among college students, affecting 30.6% of women and 25.5% of men [6], as well as other age groups [7]. Poor sleep quality and daytime sleepiness can be used as key markers for CMD [6], but it should be noted that the accuracy of sleep monitoring with consumer trackers declines with lower sleep efficiency or sleep disorders [8,9].

With the growing focus on sleep health, consumer sleep-tracking devices have seen a surge in sales, enabling the creation of new sleep databases for both research and industrial applications [10]. Traditionally, associations between sleep and health have relied on longitudinal self-report questionnaires, which are constrained by recall bias and their inability to capture the complex, dynamic evolution of sleep-related conditions. In contrast, passive data collection offers a transformative alternative. By leveraging the ubiquity of smartphones and wearable devices that continuously generate high-resolution data, it becomes possible to obtain objective, real-time, and ecologically valid assessments of sleep behavior.

For instance, Massar et al (2021) identified nocturnal sleep behavior patterns in a healthy population by integrating wearable, tappigraphy and self- report data through a clustering analysis [11]. While passive data collection via smartphone apps is convenient, its accuracy remains limited compared to that of specialized devices. A study comparing four smartphone sleep-tracking apps against polysomnography found that although all apps correlated with time in bed, only one showed a significant correlation with sleep efficiency [12]. To fully understand their potential and limitations, it is essential to investigate the behavioral factors contributing to discrepancies between smartphone-based sleep estimates and those from more accurate devices. Moreover, considering the diversity of sensors across dedicated sleep trackers and the variability in user behavior, mapping the disagreement between devices to specific behavioral patterns is a critical step.

A significant trend in 2025 is the shift toward “Smart Packages”—integrated systems that combine smartphones with one or more wearable devices [13,14]. This approach recognizes that no single device can capture the “full picture” of a patient's condition. Smartphones serve as excellent proxies for social behavior and cognitive engagement through app usage and communication logs, while wearables provide high-fidelity physiological data such as heart rate variability (HRV) and sleep architecture [13].

Central to this evolution is a radical “inversion of perspective”: the reclassification of inter-device discrepancies not as measurement noise or technical failure, but as high-value behavioral signals that provide deep insight into patient compliance, interoceptive awareness, and environmental interactions [15]. Traditionally, if a wrist-worn accelerometer and a smartphone's step counter disagreed, the difference was attributed to sensor error or placement noise. Modern frameworks, however, interpret this disagreement as a direct marker of patient behavior or clinical phenotype [15].

**Fig 1:**
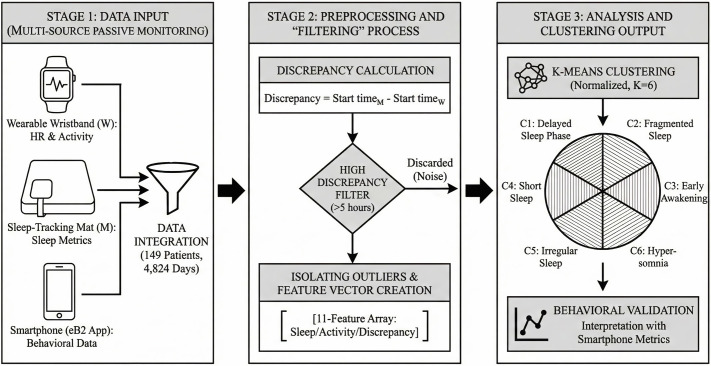
Graphical overview of the complete methodological pipeline, including data collection, preprocessing and filtering steps, and the subsequent clustering analysis.

Based on these considerations, the central hypothesis of this study is that disagreement between devices can serve as a behavioral signal rather than mere measurement noise, offering a novel means to identify abnormal sleep behavior with potential clinical relevance. Unlike prior studies combining diverse data types, this work focuses solely on discrepancies and measurements of the same variables from two tracking devices, an always on wristband and a sleep tracking mat, offering a multi-view perspective rather than a multi-source analysis. The objective is to analyze simultaneous recordings from both devices without assuming either as ground truth, and to interpret their disagreement as a behavioral signal. By isolating highly discrepant days, representing out-of-distribution samples, and validating the behavioral influence underlying these discrepancies, we identify full-day sleep behavior patterns in individuals diagnosed with CMD that are both abnormal and interpretable. Such high-discrepancy events may serve as practical indicators for monitoring treatment progress, detecting side effects, or identifying other clinically relevant phenomena associated with mental health conditions. Fig 1 shows a graphical overview of the complete analysis pipeline.

## Methods

### Study design

The current database has been acquired by means of a broader observational and prospective clinical study carried out at Hospital Universitario Fundación Jiménez Díaz (FJD) with the collaboration of Evidence Based Behavior (eB2) [16] and the Signal Theory and Communications Department of Universidad Carlos III de Madrid (UC3M). The project’s title is “Detection of physical, cognitive and emotional status changes in patients with Common Mental Disorders through monitoring and follow-up tools”. The study is encapsulated within a broader context project, IntCare-CM from the I + D REACT-UE research project in Comunidad de Madrid. The study protocol was approved by the Institutional Review Board of Fundación Jiménez Díaz in Madrid, Spain. Participants provided written informed consent before using the eB2 MindCare app, and all methods followed the ethical guidelines of the Declaration of Helsinki. No financial incentive was offered. The recruitment period was carried out from October 27^th^ (2022) to February 2^nd^ (2023). The researchers began accessing the anonymized data in February (2024).

Inclusion criteria was defined by two mayor conditions: (1) patients with 18 years or more (2) diagnosed with CMD. Only non-severe cases have been included. Participation was volunteer and informed consent was obtained during medical visits. The sample comprises 152 patients with an initial monitoring period of three months. Passive data was collected through a mobile app operating under eB2 infrastructure, and a pair of different consumer-type sleep tracking devices. At the end of the tracking phase and filtering out users with no data, a total of 4,824 days belonging to 149 patients were captured simultaneously from both devices. Only sleep data with simultaneous information from both sensors was used in this analysis.

We employed two Withings^TM^ sleep-tracking devices [17] that differ in measurement precision due to variations in their sensor configurations and functional capabilities. The wearable wristband tracker (W) functions as a medium-precision, always-on device, whereas the sleep-tracking mat (M), positioned beneath the patient’s mattress, provides higher-accuracy measurements. The specific models used were the *Pulse HR* (W) and *Sleep Analyzer* (M).

The wristband (W) relies on tri-axial accelerometer and light sensors, as well as photoplethysmography (PPG) for heart rate estimation. Its performance is partly constrained by user compliance, since the device must be worn during sleep to collect data. In contrast, the mat (M) employs microphone and pressure sensors, which yield more accurate estimations of sleep onset and offset. It is particularly effective at distinguishing true sleep from passive resting states and requires no user compliance, as it remains continuously powered and positioned under the mattress. However, the M records data only when the user is physically on the bed.

Integrating data from both devices provides a complementary, multi-view description of sleep-related behavior, rather than a unified sleep estimation model. The wristband (W) passively records body movement and physiological signals throughout the day and night, enabling the detection of sleep-like states both in and out of bed, while the sleep-tracking mat (M) records pressure- and respiration-based signals only when the user is physically in bed, providing more reliable information on bed occupancy and in-bed sleep periods. We analyze the agreement and disagreement between the independently estimated sleep sessions from each device, complemented by smartphone-derived behavioral metrics (e.g., activity, stillness, phone use) to further validate the findings. This design allows us to characterize full-day sleep-related behavior while explicitly accounting for the distinct sensing capabilities and limitations of each device.

### Sample demographics

The patient population predominantly comprised women, who accounted for 72% of the total sample. The mean age of the participants was 47 years, with a range of 18–71 years. Disorders were categorized into four groups based on their nature: anxiety disorders, major depressive disorder, impulsivity-related disorders (including adult ADHD), and others. Patients with psychotic disorders or bipolar disorder were not included. Only primary diagnoses were considered, with comorbidities not taken into account. Anxiety disorders were the most prevalent (68%), followed by major depressive disorder (15%), impulsivity-related disorders (12%), and other types of disorders (5%).

### Data preprocessing, feature selection and experimental setting

The full dataset contains recordings of 10,487 days of sleep data. Among these, 46% are filled with data from both devices, 22% with just M data, 12% with just W data, and 20% with no data.

For the clustering analysis, a filtered dataset was created, including only days with simultaneous recordings from both devices, amounting to a total of 4,824 days. Each full day of sleep was represented by five sleep-related metrics from each device, along with the start-time discrepancy between them, yielding an 11-feature array, with all dimensions expressed in hours:

1. Start time for M
2. Start time for W
3. End time for M
4. End time for W
5. Time asleep (TA) for M
6. Time asleep for W
7. Time in bed (TIB) for M
8. Time in bed for W
9. Peri-sleep time (PT): TIB minus TA for M
10. Peri-sleep time: TIB minus TA for W
11. Start-time discrepancy (difference between M and W start times)

To ensure consistent measurements, the start and end times were encoded using a scheme spanning two consecutive days, allowing the capture of both nocturnal and diurnal sleep-related activities. For each pair of measurements (start and end times) from the two devices (M and W), the reference point was set to midnight on the day the sleep data were recorded. All timestamps were then converted into hours relative to this reference, with times before midnight represented as negative values and those after midnight as positive. Finally, all features underwent min–max normalization, scaling their values to the [0, 1] range in preparation for the K-Means clustering analysis.

To quantify the agreement between simultaneous recordings, we formally define discrepancy (or device disagreement) as the difference in the sleep session start times, measured in hours. The start time corresponds to the moment a user adopts a restful position with the intention to sleep, as recorded independently by the mat and the wristband. Mathematically, this can be expressed as:


$$\text{Discrepancy }=\;{\text{Start time}}_{\text{M}}-\;{\text{Start time}}_{\text{W}}.$$

This formulation preserves the sign of the discrepancy, indicating whether one device reports an earlier or later onset than the other. Based on this definition, the dataset was filtered into three distinct discrepancy zones, representing different degrees of disagreement between devices:

1. Low discrepancy: less than 1 hour (72%; 3,491 days)
2. Medium discrepancy: between 1 and 5 hours (23%; 1,136 days)
3. High discrepancy: more than 5 hours (4%; 197 days)

Given the considerable differences in start times observed on high-discrepancy days, we sought to investigate the underlying causes of these disagreements by performing a clustering analysis focused on these outlier samples. Because the observed differences were too large to be attributed to measurement error or random noise, this approach also served to corroborate the hypothesis that such discrepancies have a behavioral origin.

However, since the thresholds used to separate low-, medium-, and high-discrepancy days were defined arbitrarily—following rational criteria—we conducted an additional clustering analysis combining medium- and high-discrepancy days. This strategy increased the available data volume, allowing for finer resolution in the learned clusters and enabling the exploration of whether high-discrepancy (outlier) patterns extended below the 5-hour threshold.

It is important to note that as the discrepancy between devices decreases, the data progressively shift toward the in-distribution region, where non-behavioral factors such as sensor variability, device usage inconsistencies, and measurement noise may infuse a greater influence on the resulting cluster composition.

Additionally, data from the eB2 application collected during the study period included several behavioral metrics: step count, stillness duration (continuous time period during which no significant movement is detected), time spent in a vehicle, app usage (user’s total spent time in applications), and phone unlock frequency. These metrics provide contextual information for interpreting sleep recordings and add complementary dimensions of patient behavior.

To incorporate these behavioral signals, we adopted a global analytical approach, computing the mean and variance of each behavioral metric within a temporal neighborhood surrounding the abnormal sleep patterns. Specifically, statistics were calculated for the day preceding, the day of, and the day following each identified abnormal cluster—thereby capturing the behavioral context before, during, and after the observed irregularity.

As a baseline for comparison and to establish quantitative references across behavioral domains, the same statistics were computed for normal days, defined as those belonging to the low-discrepancy zone.

### Clustering method

We employed the K-Means algorithm for clustering. The implementation was carried out in Python [18] using the Scikit-learn library [19], with K-Means++ initialization. This initialization method selects starting centroids based on an empirical probability distribution proportional to each point’s contribution to the overall inertia (i.e., the within-cluster sum of squared distances), which facilitates faster and more stable convergence. To determine the optimal number of clusters, we applied two quantitative evaluation criteria: inertia and the silhouette coefficient.

## Results

### Full data set overview

Overall, the sleep-related metrics are highly consistent across devices, indicating general agreement in their measurements and yielding a mean start-time discrepancy of 1.13 hours between devices. Table 1 summarizes the statistics of the 11 features derived from the 4,824 days with simultaneous recordings from both devices.

**Table 1:** Empirical Summary of sleeping statistics recorded by devices.

	Sleep Tracking Mat (M)					Wearable Wristband (W)					Discrepancy (abs)
	Start time	End time	Time asleep	Time in bed	Peri-sleep time	Start time	End time	Time asleep	Time in bed	Peri-sleep time	Start Time
Mean	23:16	08:09	7.10	8.21	1.11	23:18	08:08	7.92	8.46	0.54	1.13
Std	02:46 (h)	02:37 (h)	2.27	2.57	0.89	02:36 (h)	02:18 (h)	1.95	2.13	0.44	2.28
Min	03:22^day-1^	23:49^day-1^	0.03	0.20	0.00	01:15^day-1^	23:48^day-1^	0.00	0.01	0.00	0.00
25%	22:37	06:34	6.08	7.03	0.52	22:46	06:40	6.82	7.25	0.23	0.18
50%	23:37	07:51	7.17	8.22	0.90	23:34	07:52	7.90	8.40	0.43	0.42
75%	00:34	09:22	8.32	9.42	1.45	00:43	09:21	8.98	9.57	0.73	1.12
Max	22:37^day + 1^	23:52^day + 1^	32.23	39.38	11.32	23:43^day + 1^	23:25^day + 1^	26.62	27.85	5.80	24.98^*^
Mode	23:52	06:31	7.08	8.07	0.68	22:34	06:19	7.78	9.13	0.03	0.07

**Fig 2:**
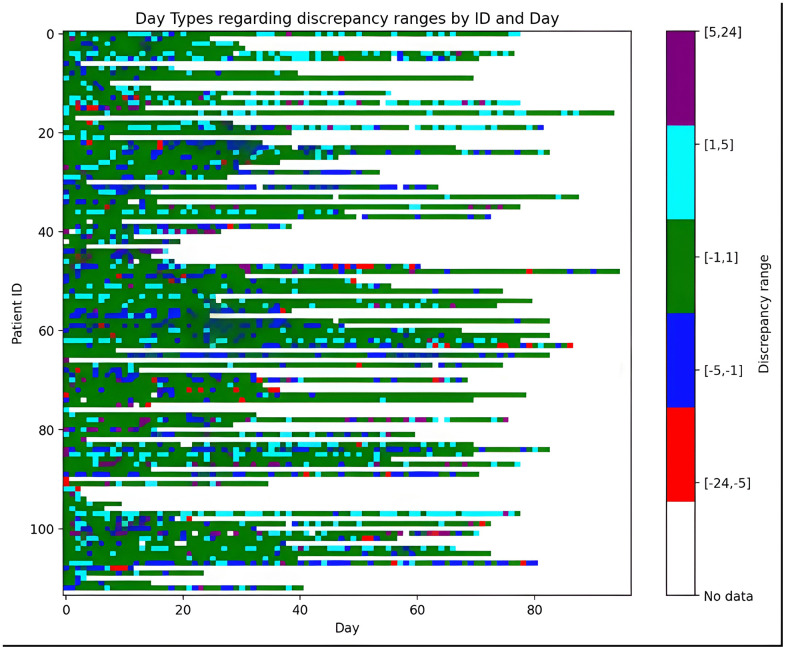
Discrepancy ranges per user (discrepancy = Start time_M_ – Start time_W_). Each row represents a user and each column a day. Color coding represents discrepancy ranges in start times taking into consideration the sign.

Fig 2 illustrates, for each individual patient, the range of discrepancies observed between the two devices. A visual inspection reveals a notable consistency in discrepancy patterns at the individual level—each patient tends to repeat the same discrepancy range throughout the observation period—suggesting a behavioral influence rather than random variation.

### High-discrepancy clustering

**Fig 3:**
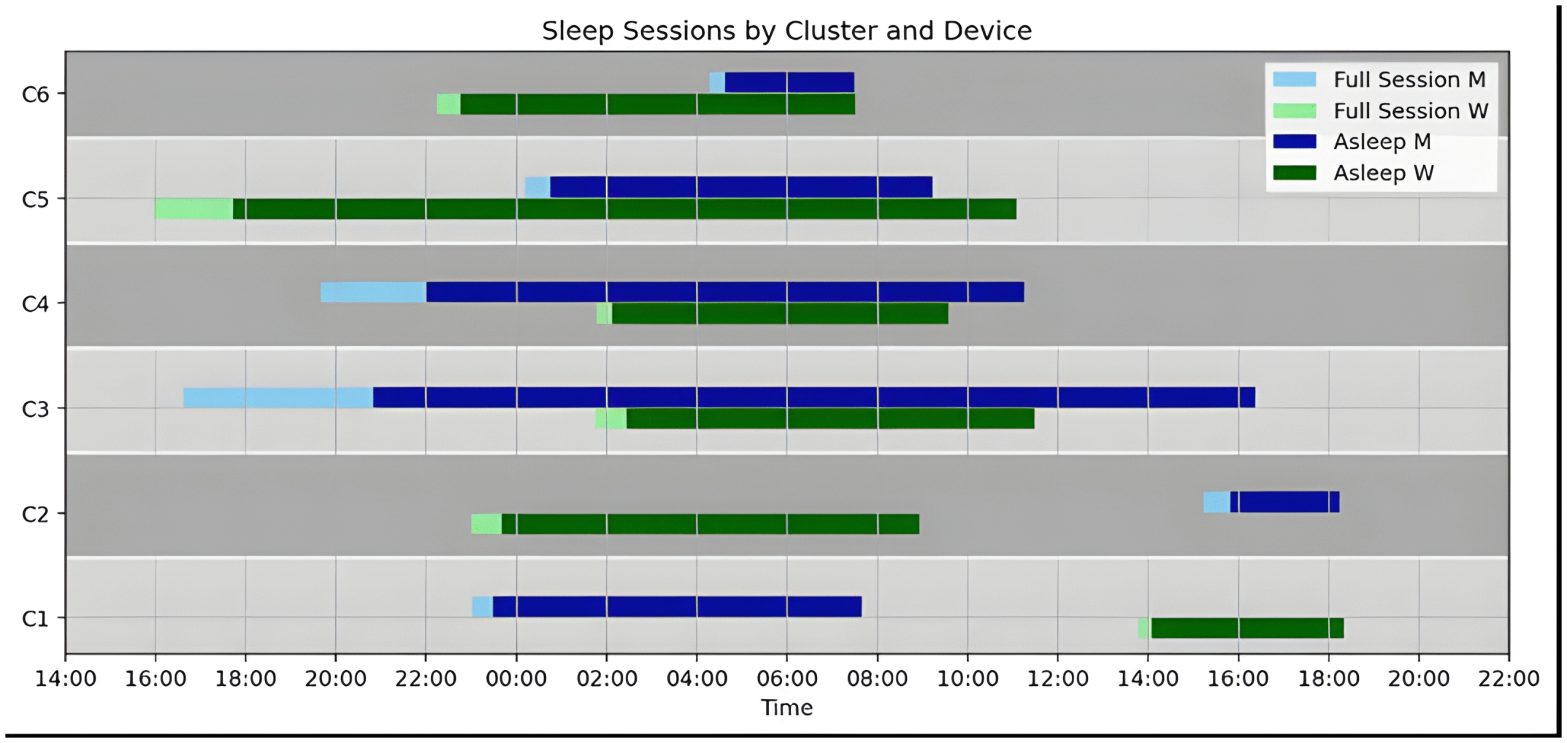
Full-day schematic of high discrepancy clusters.

The optimal number of clusters was found to be K = 6, as indicated by the silhouette coefficient and further corroborated by the elbow method applied to the inertia criterion. The delineation among these clusters was satisfactory from both quantitative and qualitative perspectives. S1 Fig (supplementary material) presents the t-SNE projection of the clustered data into a lower-dimensional space, visually demonstrating well-separated clusters. A statistical summary of each cluster is presented in Table 2, and a simplified temporal schematic of these clusters is shown in Fig 3. S2 Fig displays bar plots of the additional behavioral metrics collected through the eB2 app.

**Table 2:** Median values for sleeping metrics in high discrepancy clustering. Interquartile range (Q3-Q1) is shown in parenthesis.

Cluster	C1	C2	C3	C4	C5	C6
**Count**	15.00	41.00	15.00	33.00	40.00	53.00
**Start M**	23:01 (02:34)	15:14 (02:06)	16:37 (06:43)	19:40 (06:32)	00:11 (04:17)	04:16 (03:29)
**Start W**	13:46 (06:10)	23:00 (01:57)	01:44 (06:55)	01:47 (05:23)	15:55 (05:15)	22:14 (02:55)
**End M**	07:10 (01:37)	17:39 (03:34)	12:09 (04:03)	08:57 (02:53)	08:40 (03:08)	07:08 (02:21)
**End W**	18:01 (07:43)	08:15 (02:42)	10:47 (03:19)	09:14 (02:20)	09:16 (03:08)	06:59 (02:17)
**TA M (h)**	7.72 (2.45)	1.40 (1.58)	16.02 (8.31)	9.47 (2.92)	7.12 (2.85)	1.03 (2.40)
**TA W (h)**	3.98 (2.45)	8.50 (3.20)	8.75 (3.26)	7.60 (4.40)	12.96 (3.43)	7.93 (1.55)
**TIB M (h)**	8.38 (2.86)	2.12 (1.60)	20.77 (7.71)	12.52 (3.68)	8.02 (3.30)	1.53 (2.37)
**TIB W (h)**	4.25 (2.22)	9.32 (3.73)	9.40 (3.14)	7.93 (4.48)	15.01 (3.28)	8.68 (1.45)
**Start Disc. (h)**	−13.62 (8.95)	16.12 (4.03)	−9.22 (5.02)	−5.80 (1.48)	6.98 (2.88)	6.63 (2.72)
**PT M (h)**	0.47 (1.12)	0.58 (0.73)	4.22 (2.27)	2.30 (1.82)	0.55 (0.68)	0.35 (0.45)
**PT W (h)**	0.32 (0.59)	0.68 (0.62)	0.70 (0.66)	0.33 (0.33)	1.81 (1.06)	0.52 (0.65)

**Legend:** C1-C6 (clusters); Devices: M (sleep-tracking mat), W (sleep-tracking wristband); Sleeping Metrics: Start X (sleep onset detected by device X), End X (sleep offset detected by device X), TA X (time asleep detected by device X), TIB X (time in bed detected by device X), Start Disc. (start time discrepancy = Start M – Start W), PT X (peri-sleep time = TIB X – TA X).

We identified six distinct clusters, each representing an outlier (out-of-distribution) sleep behavior pattern:

- **Night Sleep with Afternoon Nap (C1)**: This group displays a normal nighttime sleeping session recorded by M from 23:00–7:00, with no data collection from W. Hours later, W records an afternoon nap from 13:45–18:00.

Physical activity, measured through step counts, shows a higher-than-average step count on the days preceding the behavior, compared to normal days. This is followed by a lower-than-average step count on the day when the sleep discrepancy was detected. Additionally, vehicle time increases on the days following this cluster.

- **Night Out and Afternoon Nap (C2)**: The second-largest group, comprising 41 data points, is characterized by distinct sleep sessions recorded independently by each device, similar to Cluster C1, and unlike the others that capture overlapping sessions. W detects a nighttime sleep session starting around 11:00 and ending early in the morning at about 8:15 am (median). Meanwhile, M captures a shorter afternoon sleep session lasting approximately 2 hours (TIB), typically occurring after lunch (around 3 pm) but occasionally as early as 10 am.

This behavior is characterized by the highest step count on the current and following days, along with the lowest stillness time compared to other clusters. This cluster also exhibits the highest frequency of phone unlocks, though this does not necessarily correspond to increased app usage time.

- **Extended Bed Rest, 1st case (C3)**: This cluster exhibits the longest time in bed of all groups. According to M, the patient goes to bed after lunch, around 16:30 (median), staying awake in bed for about 4 hours before falling asleep. W detects sleep onset only later, between 21:30 and 1:45 in half of the cases. Both devices show similar wake times, around 11:00. Closer inspection reveals some outliers widening the cluster's variability. Despite this, all cases share a common feature: excessive time in bed, with a median of 20.7 hours as detected by M. Moreover, W corroborates this with a minimum and maximum TIB of 6.63 and 17.48 hours, respectively. It features the lowest step count and vehicle time, alongside the highest stillness and phone usage.
- **Extended Bed Rest, 2nd case (C4)**: This cluster is characterized by the user going to bed in the late afternoon, around 20:00. M detects a long wake period before sleep onset, totaling 3.55 hours in 75% of cases. W records sleep onset later, varying between 23:18 (25%), 1:47 (median), and 4:41 (75%). Both devices show wake times around 9:00 am.

This group stands out for its peri-sleep time, like C3, where the patient delays falling asleep while maintaining a resting position in bed. Statistically, it includes sessions with longer sleep durations (median 12.53 hours recorded by M), representing another oversleeping pattern. This cluster is generally in the statistical distribution as normal days, except for a slight increase in stillness time. Additionally, vehicle time is higher on the following days compared to the previous and current days.

- **Evening Nap, Night Sleep (C5)**: W signals sleep intention around 16:00, with the patient staying awake in a resting position for about 2 hours before falling asleep. At midnight, the patient moves to bed. Both devices record similar wake times (around 9 am), with the movement to bed appearing as a sleep interruption in W's data. Like C4, this behavior follows a normal day pattern but with increased stillness time. This is accompanied by slightly elevated and continued phone usage backed up with a low frequency of unlocks.
- **Night Sleep, Early Morning Bed Move (C6)**: The largest group, with 53 days, reflects a behavior where the patient falls asleep relatively quickly around 22:00 out of bed, detected only by W. Early in the morning (4:00), the patient moves to bed and resumes sleeping. Both sessions end at approximately 7:00. This cluster is marked by increased vehicle time on the days before and after the behavior, along with lower-than-average stillness time.

We also observed clear individual consistency in abnormal sleep behaviors (S3 Fig). When examining a single participant and filtering only the high-discrepancy days, the patient consistently repeated the same sleep pattern over the 90-day period, reinforcing the notion that substantial discrepancies are behaviorally driven and reflect stable aspects of the individual’s routine.

Moreover, since clustering was performed without distinguishing between patients—and the same clusters emerged across different individuals—these recurrent patterns are not confined to single subjects but instead represent behavioral profiles that can be generalized across CMD patients.

### High- and medium- discrepancy clustering

We performed an additional K-Means clustering analysis by including both medium- and high-discrepancy recordings. Adding medium-discrepancy data reduces the influence of extremely abnormal behavior, moving the analysis toward the distribution's center where non-behavioral factors may be more important. This step helps validate the clusters identified previously and addresses the potential bias from using an arbitrary threshold to define the medium/high-discrepancy split.

This dataset partition comprised 1,333 days (27%) with simultaneous measurements. The optimal number of clusters was determined to be K = 12, as indicated by a pronounced elbow in the silhouette coefficient. S1 Table (supplementary material) summarizes the statistical characteristics of these twelve clusters, while S4 Fig presents a more schematic and interpretable visualization.

The t-SNE projection of the clusters (S5 Fig) reveals less separable groups than in the high-discrepancy case, producing a low-dimensional structure like that observed in S1 Fig, but with additional data points filling the intermediate regions between high-discrepancy recordings. Notably, clusters 5, 6, 8, 9, and 11 exhibit relatively high discrepancy values and remain spatially distant from the others, suggesting a clear distinction between normal and abnormal sleep patterns—primarily driven by pronounced disagreement between device recordings.

**Fig 4:**
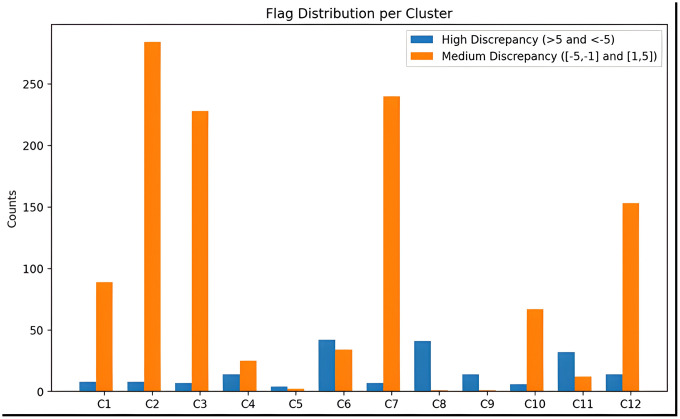
Cluster-wise distribution of medium- and high-discrepancy days. Bars show, for each of the twelve clusters, the number of days classified as high discrepancy (> 5 h or <−5 h difference between devices) and medium discrepancy (1.5–5 h or −1.5 to −5 h).

Rather than characterizing each of the twelve identified clusters individually, we analyzed the proportion of medium- and high-discrepancy days within each cluster. This approach allows us to determine which clusters represent extensions of previously identified abnormal behaviors—now appearing below the 5-hour threshold—and which correspond to more generalizable patterns involving milder discrepancies between devices. Fig 4 illustrates these proportions in a bar plot, providing a clear visualization of the distribution of medium and high discrepancies across the twelve clusters.

The identified clusters can be grouped in two main types:

- **Type 1** — **Device or sensor limitations**: Clusters C1, C2, C3, C7 and C12 fall in this category. These clusters are characterized by their general nature and high population density, predominantly comprising medium-discrepancy recordings. All clusters in this group exhibit median start-time discrepancies below approximately 2 hours, suggesting complex situations in which either the mat (M) or wristband (W) struggles to accurately detect the onset of the sleep session. In contrast, both devices tend to provide similar estimates for sleep offset times. in contrast to the offset where both M and W tend to provide similar estimates. The substantial overlap observed among these clusters indicates a shared underlying cause related to sensor limitations, with each device showing relative strengths in boundary detection under different conditions.
- **Type 2** — **Specific Abnormal Behavior**: Clusters C4, C5, C6, C8, C9, C10, and C11 are categorized as specific abnormal behaviors because they extend previously identified patterns observed on highly discrepant days, encompassing both medium- and high-discrepancy recordings. These clusters generally contain a balanced mix of medium and high-discrepancy sessions.

Despite the increased number of clusters, a consistent behavioral structure emerges at the individual level. For most patients, a single predominant behavioral pattern remains clearly dominant over the others, as illustrated in S6 Fig.

## Discussion

In this paper we have compared simultaneous recordings obtained from two consumer-grade sleep-tracking devices, using a setup that did not interfere with natural routines. Crucially, it was the discrepancy or disagreement between these devices that served as the primary signal for detecting abnormal patterns—signals that might have gone unnoticed if only a single device had been considered. This inversion of perspective enabled a deeper understanding of abnormal sleep-related behaviors.

The discrepancies observed in the current dataset are greater in both magnitude and frequency compared to those reported in previous work [11], despite the lower data quality of the sources used measuring the same sleep variables. The latent behaviors behind these discrepancies likely reflect a mix of device usage patterns, tracking limitations, and patient habits that may relate to treatment side effects, early clinical signs, or normal behaviors seen in healthy individuals.

### Clinical Hypotheses and Limitations

The clusters identified in the Results section are derived exclusively from objective, data-driven features obtained from the two sleep-tracking devices and complementary passive behavioral signals. The clinical interpretations proposed in the next subsection should therefore be understood as hypothesis-generating, rather than confirmatory. They are informed by clinical expertise and established sleep–behavior relationships in common mental disorders, but cannot be directly validated within the present dataset.

Interpretations related to oversleeping, unintended sleep onset, or treatment-related drowsiness are proposed as plausible explanations for patterns in which both devices would ordinarily be expected to record the same sleep session. However, alternative mechanisms cannot be excluded, as no concurrent clinical symptom ratings or event annotations were available.

Several limitations should be acknowledged. First, this study is observational and retrospective, which limits causal inference. Second, comorbidities, medication type, dosage, and treatment changes during the monitoring period were not explicitly modeled, despite their potential impact on sleep behavior. Finally, abnormality is defined relative to inter-device disagreement, which may also capture behaviors unrelated to the underlying condition or its treatment, such as device usage patterns or wristband charging schedules.

### Categorization of Abnormal Sleeping Patterns

High-discrepancy patterns can be categorized into three main behavioral types.

- **Type 1 — Normal night + afternoon nap**

This category includes clusters C1 (Night Sleep with Afternoon Nap) and C2 (Night Out and Afternoon Nap). Both clusters are characterized by a normal nocturnal sleep period detected by one of the devices, followed by an afternoon nap identified by the other device on the subsequent day.

In C1, the absence of nighttime data from device W suggests it may not have been worn—potentially due to charging, battery depletion, or user non-compliance. In contrast, C2 displays a nocturnal session undetected by device M, which remains constantly connected to a power source. This indicates that the user might have slept outside their bed, either intentionally (e.g., staying elsewhere overnight) or unintentionally fallen asleep outside the usual setting.

The elevated step count observed during the preceding and current days suggests increased physical activity, supporting the interpretation that the afternoon nap represents a normal compensatory rest behavior rather than a clinically relevant abnormality. These patterns are also commonly observed in the general population and therefore may not have a strong clinical association with treatment or the underlying condition.

- **Type 2 — Oversleeping behaviors**

This category encompasses clusters C3 (Extended Bed Rest, Case 1) and C4 (Extended Bed Rest, Case 2). These patterns are characterized by an early bed entry in the afternoon (typically between 4:00 p.m. and 8:00 p.m.) detected by device M, followed by an extended peri-sleep period during which the patient remains in bed but is not asleep—often accompanied by high levels of phone use activity (most prominent in C3).

Device W records the corresponding sleep session starting around 2:00 a.m., which extends into the late morning (≈10:00 a.m.) or early afternoon (≈4:00 p.m.) of the following day. Although the sleep detections between both devices show a high degree of discrepancy, the presence of the patient in bed is strongly supported by the pressure-based signal from device M, which is considered more reliable for this purpose.

This excessive time spent in bed may reflect treatment-related side effects (e.g., increased drowsiness or fatigue) or may correspond to depressive symptomatology, which is frequently associated with such behavioral patterns. This finding is further supported by the absence of high physical activity in the preceding days, in contrast to the first group.

- **Type 3 — Unintentionally falling asleep outside of bed**

This group includes clusters C5 (Evening Nap, Night Sleep) and C6 (Night Sleep, Early Morning Bed Move). These patterns are characterized by an early resting attempt in the afternoon (typically between 4:00 p.m. and 10:00 p.m.) detected by device W, outside the bed, as indicated by the absence of pressure signals from device M.

Subsequently, the patient returns to bed—around midnight in C5 or early morning in C6—where the sleep session continues until both devices register a similar offset in the morning hours (≈8:00–11:00 a.m.).

The combination of a normal step-count distribution, increased phone activity, and the transition from out-of-bed to in-bed sleep suggests that the patient fell asleep unintentionally before moving back to bed later in the night. This pattern is plausibly linked to treatment-related drowsiness or sedative side effects. Notably, these clusters have high density, indicating that this form of unintentional sleep onset is relatively common among high-discrepancy recording.

This study demonstrates the potential of integrating data from multiple consumer-grade devices to advance the understanding of sleep behavior in patients with CMD. By reframing device disagreement as an informative behavioral signal rather than measurement noise, we uncovered six robust and recurrent sleep patterns that capture not only nocturnal sleep but also peri-sleep dynamics throughout the 24-hour cycle. This multidimensional view provides a richer behavioral context than traditional single-device analyses and reveals individual-level consistency across extended monitoring periods.

From a digital health perspective, leveraging inter-device discrepancies as behavioral biomarkers offers a novel approach to remote monitoring in psychiatry. Sleep–wake irregularities and atypical rest–activity cycles have been shown to predict mood instability, cognitive decline, and relapse in patients with chronic mental disorders [20,21]. The integration of heterogeneous consumer-grade sensors enables continuous, ecologically valid tracking of these alterations in naturalistic contexts, aligning with the current shift toward personalized and measurement-based mental health care [22,23]. Recent studies demonstrate that multimodal digital phenotyping—combining actigraphy, smartphone metrics, and passive physiological signals—can detect early behavioral shifts preceding clinical deterioration, providing actionable insights for adaptive interventions [24–26]. In this sense, discrepancy-driven models may offer a complementary way to flag atypical rest–activity patterns and generate actionable hypotheses for individualized monitoring—and, in some contexts, may help anticipate clinical worsening or relapse risk.

In summary, this approach shows that combining heterogeneous sensors—each with distinct strengths and limitations—can preserve ecological validity while transforming technical discrepancies into clinically meaningful information. The resulting discrepancy-driven clusters offer new perspectives on how abnormal sleep behaviors may emerge as manifestations of treatment side effects, or routine disruptions commonly associated with CMD.s

## Supporting information

S1 Dataset Minimal Dataset and Supplementary Documentation. Includes minimal_dataset.csv, containing the primary data required to replicate the study’s findings, and README minimal dataset.docx, providing technical specifications and metadata.
(ZIP)

S1 Table Median values for sleeping metrics in medium and high discrepancy clustering. Interquartile range (Q3-Q1) is shown in parenthesis. C1-C12 (clusters); Devices: M (sleep-tracking mat), W (sleep-tracking wristband); Sleeping Metrics: Start X (sleep onset detected by device X), End X (sleep offset detected by device X), TA X (time asleep detected by device X), TIB X (time in bed detected by device X), Start Disc. (start time discrepancy = Start M – Start W), PT X (peri-sleep time = TIB X – TA X).
(DOCX)

S1 Fig t-SNE projection of high-discrepancy clusters found by K-means. (TIF)

S2 Fig Bar plots of collected passive data in close temporal vicinity for each high discrepancy cluster. Each bar represents the mean value of the data points within the respective temporal vicinity: previous day (blue), current day (orange) and posterior day (green). The orange dotted line represents the mean value for low- and medium-discrepant days. The error bars represent the standard deviation of each variable. Additional behavioral variables: Top left plot (step count), top right plot (stillness duration, in seconds), middle left plot (time spent using apps, in seconds), middle right plot (number of phone unlocks), bottom left plot (time spent in vehicles, in seconds).
(ZIP)

S3 Fig High discrepancy days for each patient with cluster assignment. (TIF)

S4 Fig Full-day schematic of medium and high discrepancy clusters. (TIF)

S5 Fig t-SNE projection of clusters found by K-means in high and medium discrepancy zone. (TIF)

S6 Fig Medium and high discrepancy days for each patient with cluster assignment. (TIF)
