# Correction: Full-day sleep pattern analysis in common mental disorders: Leveraging highly discrepant recordings from two consumer tracking devices

**DOI:** 10.1371/journal.pone.0349346

**Published:** 2026-05-12

**Authors:** Óscar Jiménez Rama, Antonio Artés, Enrique Baca-García, Jorge López-Castromán

The following information is missing from the Funding statement: This work was supported through the R&D activities program with reference TEC-2024/COM-89 and acronym IDEA-CM, granted by the Community of Madrid through the Directorate-General for Research and Technological Innovation under Order 2402/2024, dated May 31. Additionally, this work was supported by the Industrial Doctorates Programme of the Community of Madrid under project IND2022/TIC-23550. AAR is part of the ELLIS Unit Madrid (European Laboratory for Learning and Intelligent Systems) funded by the Community of Madrid.
