# Supplementary material for: Full-day sleep pattern analysis in common mental disorders: Leveraging highly discrepant recordings from two consumer tracking devices
Source: PLoS One. 2026 Apr 9;21(4):e0346876. doi: 10.1371/journal.pone.0346876 (PMC13065001; doi:10.1371/journal.pone.0346876)
Supplement: S1 Dataset — Includes minimal_dataset.csv, containing the primary data required to replicate the study’s findings, and README minimal dataset.docx, providing technical specifications and metadata. (ZIP) [file pone.0346876.s001.zip › S1 Dataset/README_minimal_dataset.docx]

**README — Minimal Dataset**

*Full-day sleep pattern analysis in common mental disorders: leveraging highly discrepant recordings from two consumer tracking devices*

# **1. Overview**

This file describes the structure, content, and encoding conventions of the minimal dataset provided as Supporting Information for the above-referenced manuscript published in PLOS ONE.

The dataset contains anonymized sleep metrics derived from two consumer-grade tracking devices used in a public-private clinical trial. No personally identifiable information is included. Calendar dates have been deliberately withheld to protect participant privacy.

# **2. File**

- Filename: minimal_dataset.csv
- Format: Comma-separated values (CSV), UTF-8 encoding
- Rows: 4,824 (each row represents one night of sleep for one participant)
- Columns: 8 (see Section 4 for full descriptions)

# **3. Devices**

Data were collected simultaneously from two consumer tracking devices. Column names use a suffix to identify the source device:

- _M — Mattress-based sleep tracker (under-mattress sensor)
- _W — Wristband-based sleep tracker (wrist-worn actigraphy device)

Both devices recorded the same sleep episodes independently. The discrepancies between their measurements are the primary subject of analysis in the manuscript.

# **4. Time Encoding Convention**

All time variables (Start and End) are expressed in decimal hours using a midnight-anchor reference system:

**Midnight = 0.0**

Under this convention:

- Negative values indicate times before midnight (e.g., −0.5 = 23:30, −1.0 = 23:00)
- Positive values indicate times after midnight (e.g., 7.0 = 07:00, 8.5 = 08:30)
- Fractional hours represent minutes as decimal fractions (e.g., 6.25 = 06:15)

This encoding preserves the mathematical structure of sleep timing (allowing computation of means, standard deviations, and differences across participants) while preventing the recovery of any specific calendar date or participant identity.

Duration variables (TA and TIB) are expressed as positive decimal hours measured from sleep onset, not anchored to midnight.

# **5. Data Dictionary**

| **Column Name** | **Unit** | **Short Description** | **Full Description** |
| --- | --- | --- | --- |
| Start_M | Decimal hours | Sleep onset time — Mattress | Time at which the mattress device detected sleep onset. Encoded relative to midnight (0.0). Negative values indicate sleep onset before midnight; positive values indicate onset after midnight. |
| Start_W | Decimal hours | Sleep onset time — Wristband | Time at which the wristband device detected sleep onset. Same encoding as Start_M. |
| End_M | Decimal hours | Sleep offset time — Mattress | Time at which the mattress device detected sleep offset (wake-up). Encoded relative to midnight (0.0). Values above 0 indicate wake-up after midnight. |
| End_W | Decimal hours | Sleep offset time — Wristband | Time at which the wristband device detected sleep offset. Same encoding as End_M. |
| TA_M | Decimal hours | Total sleep time — Mattress | Total time asleep as estimated by the mattress device. Computed as the duration of consolidated sleep within the sleep window. |
| TA_W | Decimal hours | Total sleep time — Wristband | Total time asleep as estimated by the wristband device. |
| TIB_M | Decimal hours | Time in bed — Mattress | Total time spent in bed as recorded by the mattress device, from first detection of lying down to final detection of leaving bed. |
| TIB_W | Decimal hours | Time in bed — Wristband | Total time spent in bed as recorded by the wristband device. |

# **6. Ethical and Privacy Considerations**

This dataset was collected as part of a public-private clinical trial conducted at Hospital Universitario Fundación Jiménez Díaz with the collaboration of Evidence Based Behavior (eB2) and the Signal Theory and Communications Department of Universidad Carlos III de Madrid (UC3M). The dataset shared here constitutes only the sleep metric variables, which were determined to be insufficient for re-identification or personal data explotation of participants. Behavioral time-series data (physical activity, phone usage, mobility) are not included and are subject to access restrictions. To request access to the behavioral time-series data contact Miguel Madueño with email [[miguel.madueno@eb2.tech](mailto:%22Miguel%20Madue%C3%B1o%20Sanz%22%20%3Cmiguel.madueno%40eb2.tech%3E)].

# **7. Citation**

If you use this dataset, please cite the associated manuscript:

*Jiménez Rama, O. et al. Full-day sleep pattern analysis in common mental disorders: leveraging highly discrepant recordings from two consumer tracking devices. PLOS ONE.*

# **8. Contact**

For questions about this dataset, please contact the corresponding author via the journal's published contact information.
