# Supplementary material for: Full-day sleep pattern analysis in common mental disorders: Leveraging highly discrepant recordings from two consumer tracking devices
Source: PLoS One. 2026 Apr 9;21(4):e0346876. doi: 10.1371/journal.pone.0346876 (PMC13065001; doi:10.1371/journal.pone.0346876)
Supplement: S1 Table — Interquartile range (Q3-Q1) is shown in parenthesis. C1-C12 (clusters); Devices: M (sleep-tracking mat), W (sleep-tracking wristband); Sleeping Metrics: Start X (sleep onset detected by device X), End X (sleep offset detected by device X), TA X (time asleep detected by device X), TIB X (time in bed detected by device X), Start Disc. (start time discrepancy = Start M – Start W), PT X (peri-sleep time = TIB X – TA X). (DOCX) [file pone.0346876.s002.docx]

Table S1

**Table S1. Median values for sleeping metrics in medium and high discrepancy clustering. Interquartile range (Q3-Q1) is shown in parenthesis.**

| Cluster | C1 | C2 | C3 | C4 | C5 | C6 |
| --- | --- | --- | --- | --- | --- | --- |
| Count | 97.00 | 292.00 | 235.00 | 39.00 | 6.00 | 76.00 |
| Start M | 01:11 (02:54) | 23:12 (01:22) | 23:45 (01:45) | 19:23 (05:13) | 22:01 (01:36) | 03:51 (03:23) |
| Start W | 03:17 (02:36) | 21:45 (01:16) | 22:03 (01:43) | 23:18 (04:04) | 14:23 (21:15) | 22:54 (02:11) |
| End M | 11:00 (01:45) | 06:30 (01:18) | 08:47 (01:29) | 12:07 (03:44) | 12:14 (09:57) | 06:57 (01:53) |
| End W | 10:51 (01:32) | 06:32 (01:19) | 08:52 (01:37) | 11:16 (02:38) | 09:52 (04:44) | 07:27 (01:34) |
| TA M (h) | 7.77 (3.07) | 6.48 (1.40) | 7.78 (1.69) | 12.47 (4.66) | 25.57 (1.49) | 1.57 (2.61) |
| TA W (h) | 7.37 (2.10) | 8.07 (1.33) | 9.88 (1.58) | 10.42 (2.91) | 7.60 (4.29) | 7.90 (1.46) |
| TIB M (h) | 9.40 (2.52) | 7.11 (1.50) | 8.60 (1.46) | 16.65 (4.23) | 30.98 (7.87) | 2.13 (2.61) |
| TIB W (h) | 7.75 (2.07) | 8.84 (1.41) | 10.77 (1.48) | 11.98 (3.27) | 8.21 (4.30) | 8.27 (1.60) |
| Start Disc. (h) | -1.82 (1.72) | 1.44 (0.85) | 1.68 (1.10) | -4.12 (4.34) | -16.57 (18.95) | 5.24 (3.74) |
| PT M (h) | 1.33 (1.03) | 0.57 (0.55) | 0.75 (0.74) | 3.97 (1.94) | 5.19 (2.49) | 0.40 (0.62) |
| PT W (h) | 0.27 (0.30) | 0.75 (0.52) | 0.90 (0.63) | 1.15 (0.74) | 0.56 (0.14) | 0.47 (0.62) |

| Cluster | C7 | C8 | C9 | C10 | C11 | C12 |
| --- | --- | --- | --- | --- | --- | --- |
| Count | 247.00 | 42.00 | 15.00 | 73.00 | 44.00 | 167.00 |
| Start M | 23:10 (01:53) | 15:15 (02:06) | 22:47 (02:49) | 00:07 (02:02) | 23:36 (03:49) | 21:48 (01:37) |
| Start W | 01:23 (02:34) | 23:00 (02:02) | 13:47 (04:41) | 22:52 (02:12) | 17:01 (05:31) | 23:42 (01:38) |
| End M | 07:37 (01:40) | 17:43 (03:37) | 07:10 (01:37) | 02:54 (02:13) | 08:01 (03:36) | 08:58 (01:35) |
| End W | 07:20 (01:38) | 08:04 (02:42) | 19:14 (06:23) | 07:38 (03:47) | 10:01 (04:04) | 08:45 (01:50) |
| TA M (h) | 6.40 (1.88) | 1.39 (1.58) | 8.17 (2.17) | 1.68 (2.02) | 7.22 (3.60) | 8.63 (1.98) |
| TA W (h) | 5.37 (1.90) | 8.44 (3.58) | 3.98 (2.39) | 7.72 (2.52) | 13.57 (2.50) | 8.42 (1.49) |
| TIB M (h) | 8.10 (1.63) | 2.01 (1.60) | 9.28 (2.22) | 2.65 (1.95) | 8.07 (3.82) | 10.88 (2.02) |
| TIB W (h) | 5.80 (1.85) | 9.25 (4.15) | 4.25 (2.13) | 8.35 (3.07) | 15.49 (2.18) | 8.88 (1.73) |
| Start Disc. (h) | -2.07 (1.71) | 15.80 (3.96) | -14.98 (7.85) | 1.38 (3.60) | 6.68 (4.38) | -2.13 (1.58) |
| PT M (h) | 1.47 (0.92) | 0.61 (0.69) | 0.62 (1.10) | 0.70 (1.00) | 0.63 (0.84) | 2.37 (1.26) |
| PT W (h) | 0.30 (0.33) | 0.67 (0.64) | 0.20 (0.46) | 0.73 (0.63) | 2.05 (1.04) | 0.45 (0.52) |

**Legend:**

C1-C12 (clusters); Devices: M (sleep-tracking mat), W (sleep-tracking wristband); Sleeping Metrics: Start X (sleep onset detected by device X), End X (sleep offset detected by device X), TA X (time asleep detected by device X), TIB X (time in bed detected by device X), Start Disc. (start time discrepancy = Start M – Start W), PT X (peri-sleep time = TIB X – TA X).
